# Malathion-Induced Hematoxicity and Its Recovery Pattern in *Barbonymus gonionotus*

**DOI:** 10.1155/2021/9417380

**Published:** 2021-12-21

**Authors:** Cynthia E. Mrong, Md. R. Islam, Kamrunnaher Kole, Nusrat N. Neepa, Md. J. Alam, Md. R. Haque, Umme O. Rahman, Golam M. Mostakim

**Affiliations:** ^1^Department of Fisheries Biology and Aquatic Environment, Bangabandhu Sheikh Mujibur Rahman Agricultural University, Gazipur 1706, Bangladesh; ^2^Department of Aquaculture, Bangabandhu Sheikh Mujibur Rahman Agricultural University, Gazipur 1706, Bangladesh; ^3^Department of Biochemistry and Molecular Biology, Bangladesh Agricultural University, Mymensingh 2202, Bangladesh; ^4^Department of Fisheries Biology and Genetics, Bangladesh Agricultural University, Mymensingh 2202, Bangladesh; ^5^Graduate Training Institute, Bangladesh Agricultural University, Mymensingh 2202, Bangladesh

## Abstract

An experiment was conducted to assess malathion-induced hematological responses of *Barbonymus gonionotus* (silver barb) and its recovery patterns in malathion-free water. Fish (45 days old) were exposed to two sublethal concentrations, namely, 25% and 50% (i.e., 3.78 and 7.56 ppm) of LC_50_ (15.13 ppm) of malathion for 28 days, followed by a postexposure recovery period for the same time. The hematological parameters were examined after 1, 7, 14, 21, and 28 days of exposure as well as after the postexposure recovery time. Except in the case of the control group (0% of malathion), the obtained results revealed that malathion exposure resulted in significantly (*p* < 0.05) higher prevalence and severity of micronucleus and lower values of Hb, PCV, and RBC and significantly higher values of WBC in a concentration- and time-dependent manner. The values of blood glucose, MCV, MCH, and MCHC showed mixed trends during the experiment. During the recovery period, all blood parameters (micronucleus, glucose, Hb, PCV, RBC, WBC, MCV, MCH, and MCHC) partially recovered, which means that the recovery period was not long enough for the organisms to recover from the previous exposure. The study thus confirms that hematology is a sensitive indicator for fish to detect toxicity caused by different chemicals. Changes in these parameters can provide useful information about environmental conditions and risk assessment of aquatic organisms.

## 1. Introduction

The use of pesticides has become an inevitable feature of agriculture for pest control [1]. It is assumed that 40% of the crop loss can be caused by pests and insects, which is a substantial loss [2]. Pesticide contributes to enhancing crop production but at the same time pollutes the aquatic environment [3]. Pesticides from agricultural fields find their way into the natural water bodies through surface runoff and adversely affect the quality of water surfaces [4]. Pesticides exposure causes toxicity in many aquatic organisms, and fish is the most common among them [5]. Pesticides enter mainly through the gills or epithelial tissues, hence into the blood, and subsequently to different organs or systems. If the toxin reaches the fish's body, it can damage the organs, causing physiological and pathological problems [6].

About two million tons of pesticides are currently utilized annually [7]. Among the major groups of pesticides (insecticides), organophosphorus is the widely used class of pesticides [8]. Recently, organophosphorus pesticides (OPs) replaced organochlorine pesticides (OCs) in agriculture due to their small number of degradation and residues. However, such pesticides are extremely toxic to aquatic organisms and can have severe and long-term contamination effects on nontarget aquatic species such as fish [9]. Commonly used organophosphorus pesticides are malathion, methyl parathion, dichlorvos, trichlorfon, chlorpyrifos, diazinon, fenitrothion, quinalphos, phosphamidon, and so on [8]. Many reports have indicated that malathion, even at low concentrations, harms ﬁsh [10]. Therefore, monitoring the impact of these pesticides is essential.

Malathion (O,O-dimethyl dithiophosphate of diethyl mercaptosuccinate) belongs to the chemical family organophosphates. It is used in controlling pests in agricultural fields and controlling mosquito and fruit fly eradication programs. Malathion is also found in shampoos that are used for treating lice. Malathion is poisonous when it comes into touch with the skin, ingested, or inhaled [11]. It changed the metabolic activity of the fish, causing them to die [12]. Because fish is an important protein source for humans, it is essential to investigate the hazardous effects of pesticides on fish.

Understanding the adaptation and recovery is an important tool in the field of risk assessment [13]. In fish, a blood biochemistry test indicates what is happening in the body of fish exposed to pesticides. In some cases, the intensity of the damage due to exposure to pesticides can be recognized by abnormality levels in some biochemical parameters in the blood [14]. Therefore, the hematological study is considered a useful diagnostic tool to explore physiological and metabolic alterations [15].

Micronuclei, which are extranuclear bodies of chromosome fragments, also reflect the physical and chemical alterations due to pesticides. Fish micronuclei have proven to be a useful tool for determining genotoxic properties (DNA damage or genetic alteration) of compounds subsistent in the aquatic environment [16, 17].

However, the study on the recovery of malathion-treated fish on certain parameters is very limited. Therefore, the aim of the present investigation was to assess the hematological changes in *B. gonionotus* following the exposure of malathion and recovery pattern after withdrawal of exposure.

## 2. Materials and Methods

### 2.1. Experiment 1: Toxicity Assay

The acute toxicity test on *B. gonionotus* with organophosphorus pesticide malathion followed the OECD Directive No. 203 Fish, acute toxicity test [18].

### 2.2. Collection and Rearing of Experimental Fish

Healthy and uniform-sized juveniles of *B. gonionotus* (45 days old) were obtained from a local fish hatchery. Fish with an average weight of 4.85 ± 0.20 g and a standard length of 6.92 ± 0.64 cm were selected. They were acclimatized to laboratory conditions for 14 days. The random allocations of 480 individuals were done and transferred into a cemented tank retaining dechlorinated tap water oxygenated through an aeration system. Fish were fed twice (9 am and 5 pm) a day with commercial dry pellets containing 38% protein. The semirenewal of tank water was accomplished every alternative two days. When the mortality rate was less than 1%, hence the fish were considered well adapted.

### 2.3. Preparation of Test Solution

The commercial-grade (25% EC with 57% purity) liquid malathion collected from the authorized dealer was measured by a micropipette (Model E-MILB5700, England). The test water was kept in the tank for 24 h before malathion was added. To make the test solution, desired pesticide concentrations were carefully poured into 30 L of dechlorinated tap water. Then to ensure full mixing, the solution was gently stirred with a glass rod.

### 2.4. Static Bioassays

Prior to the study, environmental concentrations [19] and previous findings on fish exposed to malathion [20] were reviewed. Before starting the test, all the glass aquaria were cleaned and filled with dechlorinated tap water. For range finding, ten juvenile fish were released into a fish tank containing 30 L of water and sequential concentrations (2.00, 6.00, 8.00, 10.00, and 30.00 ppm) of malathion. Mortalities were recorded (24, 48, 72, and 96 h), and dead fish were removed immediately.

To assess the lethal concentration value (LC_10–100_) of malathion, a static acute toxicity bioassay was used. In the basic test, there were nine groups (seven test groups and two control groups) with replicates. Different concentrations were provided to each group like 7.5, 10.50, 13.00, 15.00, 18.50, 20.00, and 25.00 ppm for 96 h. Control fish were kept in dechlorinated tap water that was free of malathion. In order to obtain a homogeneous concentration of the toxic compound, aeration for 2 h was applied to the aquarium (60 cm × 45 cm × 45 cm), and randomly ten fish were then transferred to each test aquarium. Twenty-four hours before the experiment, feeding was stopped. Fish were considered dead when visible movement (e.g., operculum movements) ceased, and there was no response to gentle probing of the caudal peduncle. Dead fish were removed as soon as possible, and the mortality rate was registered. Probit analysis was used to calculate the LC_10_, LC_50_, and LC_100_ values for the various time interval [21].

### 2.5. Experiment 2: Sublethal Test of Malathion on Fish

Based on the 96 h LC_50_ of malathion pesticide, 120 silver barb fish were exposed for 4 weeks to the nominal concentrations of 25% (3.78 ppm) and 50% (7.56 ppm) value of the LC_50_ (15.13 ppm) of the malathion. There were triplicates and a control group for each concentration. The fish were fed twice (9 am in the morning and 5 pm in the afternoon) a pelleted diet with a crude protein content of 38% and a body weight of 3%. The toxicant and test water were renewed at two-day intervals to keep the toxicant's concentration steady, the level of dissolved oxygen, and to keep the amount of ammonia in the experiment as low as possible. Three fish were sampled from control and the concentrations at 7 days' intervals until the end of 4 weeks. No mortalities occurred in any group during the experimental period.

### 2.6. Assessment of Hematological Parameters

For hematological assessment, blood samples of three fish from each test aquarium were collected after the fish were exposed to malathion compounds. Blood samples were taken from each fish's caudal vein. To avoid stress effects, the whole blood withdrawal process took less than 1 min per fish. Collected blood was forced gently into sterilized Eppendorf tubes containing anticoagulant (ethylene diamine tetra-acetic acid, EDTA) to give a final concentration of 5 mg EDTA per cm^3^ blood. Blood samples were gently mixed and discarded if clots were discovered in the vial during inspection at the laboratory.

A digital blood glucose kit (GLUCOLAB) was used to measure blood glucose levels. Three fish were randomly selected from each treatment, and blood samples were taken at 1, 7, 14, 21, and 28 days. A drop of blood sample was placed on strips connected to the GLUCOLAB autocoding blood glucose test meter, and results were recorded. The values were expressed in mg/dl. Hemoglobin (Hb) estimation was done by using a digital EasyLife Hb meter. The values were expressed in g/dL.

Blood glucose, packed cell volume (PCV), hemoglobin (Hb), white blood cells (WBC), and red blood cells (RBC) were determined using the method of [22]. Mean corpuscular volume (MCV), mean corpuscular hemoglobin (MCH), and mean corpuscular hemoglobin concentration (MCHC) were calculated using the following formula:

(1) $$\begin{array}{c} \text{MCHC}=\left(\frac{\text{Hb}}{\text{PCV}}\right)\times 100,\\ \text{MCV}=\left(\frac{\text{PCV}}{\text{RBC}}\right)\times 10,\\ \text{MCH}\left(\text{pg}\right)=\frac{\left(\text{Hb}\times 10\right)}{\text{RBC}}\text{.} \end{array}$$

### 2.7. Measurement of Micronucleus

On every sampling day, fish were collected from aquaria, and later slime and water present on the external surface of the fish were wiped using blotting paper. Blood samples were collected from at least 3 fish of each tank with heparinized syringes from the posterior part of the body, spread onto medical glass slides, and dried at room temperature for 10 min. The spread was settled with methanol for 10 min and dyed with 5% Giemsa stain and consequently cleansed with tap water. The glass slides were dehydrated at room temperature and after that mounted with DPX and viewed beneath a microscope (MICROS, MCX100) using the X100 lens. A minimum of three glass slides were set up for every individual fish, and 2,000 cells were counted from each glass slide with no less than five fish investigated in every gathering. At the best cell with untarnished cellular and nuclear films, coding and blind scoring were done.

### 2.8. Experiment 3: Recovery Pattern of Fish Exposed to Malathion 25EC

After exposure to 25% and 50% of LC_50_ malathion for 28 days, treated fish were transferred without stress to malathion-free freshwater tanks for recovery observations. The water was changed, and the fish were fed every day in the meantime (9 am and 5 pm). Samplings were done at a 7-day interval (i.e., 1, 7, 14, 21, and 28 d). During the experimentation, feeding of fish was done fresh after changing water on an alternate day. At least at the end of days 1, 7, 14, 21, and 28 after exposures, at least 3 fish from each aquarium were collected for the same parameters sampled earlier and their recovery assessed. A continuous aeration system was applied for sufficient dissolved oxygen.

### 2.9. Water Quality Parameters

Water temperature and water chemistry characteristics of dissolved oxygen and pH were monitored according to (American Public Health Association) [23] in all test tanks every day. The temperature was measured by a thermometer, and dissolved oxygen and pH were measured by DO meter (Model 2020, UK) and pH meter (Model YSI 58, USA), respectively.

### 2.10. Statistical Analysis

The normality (Shapiro-Wilk test) and homogeneity (Levene's test) of variance test were performed before any statistical analysis in all groups of data (MN, Hematological contents). Statistical analyses were performed using two-way analysis of variance (ANOVA) at a significant rate of *p* < 0.05. A post hoc test was carried out using Duncan's multiple comparison procedure. All the data have been expressed in mean ± SD. All statistics were carried out using SPSS 16.0 version.

## 3. Results

### 3.1. Bioassay Study of Median Concentration (LC_50_)

**Figure 1:**
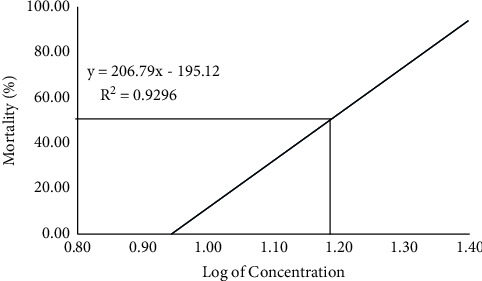
Graph showing linear transformation and the relationship of a probit of log concentration of profenofos used to determine LC_50_.

Based on the range finding test (2 to 30 ppm) of malathion, the median lethal concentration (LC_50_) of malathion for *B. gonionotus* was found to be between 10 and 30 ppm. The median lethal toxicity analysis was conducted for the concentration of malathion ranging from 7.50 to 25.00 ppm. The study showed that practically there was no mortality noticed up to a concentration of 10 ppm. Fish exposed to 96 h of malathion at a concentration of 10.50 ppm showed 10% mortality, while at a concentration of 25.00 ppm, 100% mortality was noticed in 96 h. According to the probit study, the lethal concentration for 50% mortality of the fish at 96 h was 15.13 ppm (Figure 1).

### 3.2. Effects of Malathion on Micronucleus (MN) Formation

**Figure 2:**
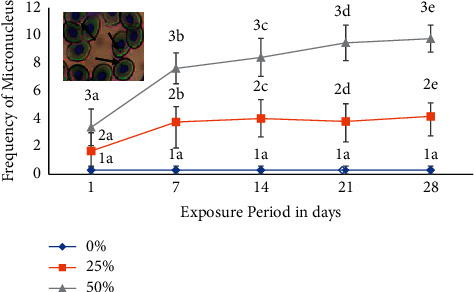
Frequency of micronuclei (MN) in the erythrocytes of silver barb exposed to different sublethal concentrations of malathion. Values with different numeric superscripts differ significantly (*p* < 0.05) between concentrations within a duration in MN. Values with different letter superscripts differ significantly (*p* < 0.05) between duration within concentration in MN. All values are expressed as mean ± SD. The photograph in the inset shows MN (arrow).

In pesticide-free water, the rate of micronuclei was nearly zero (0.31 ± 0.29), but a statistically significant increase (*p* < 0.05) in the frequency of MN was noted in a concentration- and duration-dependent manner in fish exposed to sublethal concentrations 25% and 50% of LC_50_ of malathion with respect to the corresponding control. The frequency of MN was 1.37 ± 1.15, 3.46 ± 1.87, 3.71 ± 1.32, 3.5 ± 1.47, and 3.87 ± 1.41 for 1, 7, 14, 21, and 28 days, respectively, at 25% concentration. Similarly, at 50% concentrations, micronuclei abnormality was 1.71 ± 1.32, 3.87 ± 1.11, 4.41 ± 1.37, 5.67 ± 1.28, and 5.60 ± 0.97 for 1, 7, 14, 21, and 28 days of exposure, respectively. About a three- to fivefold increase in the frequency of MN was noted at higher-concentration exposures indicating the genotoxic effects of pesticide (Figure 2).

### 3.3. Effects of Malathion on Hematological Parameters

**Figure 3:**
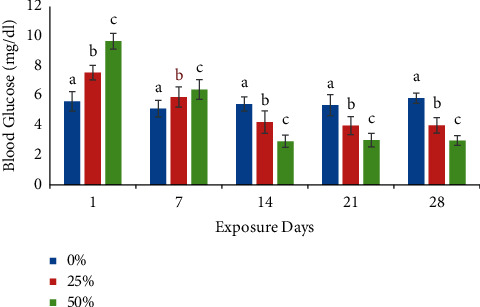
Effects of sublethal exposure of malathion on glucose level (mean ± SD) at different time intervals in silver barb. Different superscript letters are significantly different at *p* < 0.05.

Blood biochemical and hematologic parameters were assessed up to 28 days after the start of exposure to various sublethal doses of malathion in this study. According to our result, the blood glucose level was significantly higher on day 1 and decreased at 7, 14, 21, and 28 days of exposure periods compared to the control group (Figure 3).

The mean Hb, PCV, RBC, WBC, and derived erythrocyte indices (MCV, MCH, and MCHC) of silver barb exposed to chronic toxicity of malathion are presented in Table 1. The alterations observed in hematologic parameters were significantly different (*p* < 0.05) compared to the control. A significant difference (*p* < 0.05) were also observed between the various hematologic parameters with different concentrations and exposure period of the toxicant. The Hb, PCV, and RBC significantly decreased, but WBC significantly increased during toxicity of malathion at 1, 7, 14, 21, and 28 days of exposure periods in both concentrations (T2 and T3) compared to the control group (T1). Other hematologic indices, including MCV, MCH, and MCHC, showed a biphasic trend during the exposure period of malathion.

### 3.4. Recovery Responses of MN Induced by Malathion

**Figure 4:**
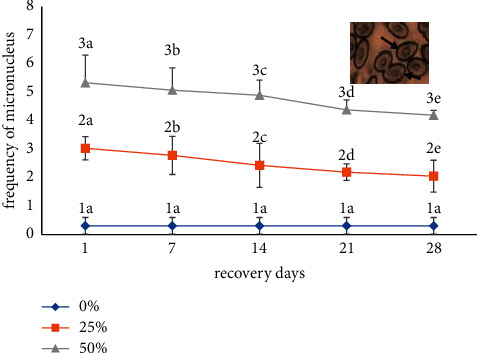
Recovery responses of MN induced by malathion (mean ± SD) at different time intervals in *B. gonionotus*. Values with different numeric superscripts differ significantly (*p* < 0.05) between concentrations within a duration in MN recovery. Values with different letter superscripts differ significantly (*p* < 0.05) between duration within concentration in MN recovery. All values are expressed as mean ± SD. The photograph in the inset shows MN recovery (arrow).

The data of the recovery responses of MN are presented in Figure 4. The MN content of the fish in the recovery period is low, and its value has a statistically significant change (*p* < 0.05). Almost all anomalous nuclei are normalized, and the recovery rate of the 25% concentration of the malathion shows a higher recovery rate than that of the concentration of 50%. The experimental results show that the recovery rate of micronuclei formation is the highest at 28 days at a concentration of 25% (Figure 4).

### 3.5. Hematological Parameters after Recovery Assessment

**Figure 5:**
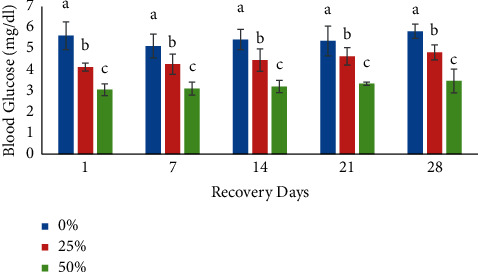
Glucose level (mean ± SD) at different time intervals in malathion-free water during recovery experiment. Different superscript letters are significantly different at *p* < 0.05.

Investigations on recovery were performed by transferring fish to pesticide-free water for a period of 28 days (Table 2). During the recovery period, glucose (Figure 5), Hb, HCT, RBC, MCV, and MCH levels were increased, whereas WBC count was decreased when compared to the 28-day exposure period of malathion pesticide (Table 2). The MCHC values showed either an increased or decreased result. The recovery of hematologic profiles after exposure indicates that malathion entering the system cannot be concentrated in the body and is slowly removed, leading to recovery.

## 4. Discussion

LC_50_ of the organophosphorus pesticide, malathion, was found to be 15.13 ppm for *B. gonionotus* which is slightly toxic as reported by EPA [24]. The LC_50_ value of malathion in the freshwater fish *Ophiocephalus punctatus* and *Tilapia mossambica* was found to be 16 ppm and 5.5 ppm, respectively [9, 25]. Again the 96 h LC_50_ values of the insecticide deltamethrin for *Heteropneustes fossilis* were 1.86 ppm [26]. The difference in the LC_50_ values is due to its dependency upon various factors, namely, species, size, and sensitivity to the toxicants, concentration, and duration of exposure [27].

In our findings, an increment in the frequency of micronuclei in silver barb's erythrocyte was explicit after 28-day exposure of pesticide on concentration and duration of exposure of malathion. The incidence of micronuclei (genome damage events) generation is a biomarker of genotoxic events and chromosomal instability or damage [28] and could intensify the risk of developmental and degenerative diseases in fish following exposure to cytotoxic and/or genotoxic agents [29, 30]. Micronuclei were also investigated to initiate during chromatid fragments caused by the disrepair of DNA breaks or anaphase from a lagging acentric chromosome or unrepaired DNA breaks [31]. Therefore, MN in the present study showed that extensive use of pesticides can cause genetic damage in the silver barb.

An advantage of testing pesticides in the freshwater ecosystem is that this type of research may provide information on the recovery of the system after pesticide contamination has stopped. A 28-day recuperation period demonstrated the foremost astounding recuperation reaction, and also the decreasing micronuclei arrangement could indicate repair of damaged DNA, a great degree of intensely injured cells, or both [32, 33]. This reverse connection between the time of application and DNA harm could be attributable to the danger of xenobiotics that disturb the enzymatic process in the formation of DNA damage [34]. Another speculated mechanism is that metabolic enzymes (such as cytochrome P450) in various tissues are activated by genes, offering a protective mechanism against harmful drugs [35]. A similar healing process was observed in isolated human lymphocytes and fish for malathion-like pesticides [36]. Other parameters, such as DNA repair capacity and cell removal kinetics, can also help to eradicate damaged tissues, which might be linked to spontaneous MN and nuclear deformities frequency interspecies differences [14]. Furthermore, enhanced erythropoiesis may dilute or conceal the number of MN, and cell cycle and maturation time must be taken into account [37].

Present findings showed that malathion had some effect on the hematologic parameters of *B. gonionotus*. For any chemical pollutant, like pesticides, blood glucose has been shown to be a sensitive measure of environmental stress [14]. In the present findings, increased glucose levels in the malathion exposed fish might be due to the enhanced gluconeogenesis response of stressed fish in their effort to meet their new energy demands [38] for combating the stress induced on the fish by malathion. Increased glucogenesis and glycogenolysis may have contributed to the observed rise in glucose levels as well as inhibition of glycogenolysis and glycogenesis during stress [39]. These findings are similar to the responses of other fish treated with organophosphorus pesticides such as grass carp exposed to endosulfan [40]. During the study period, glucose level was found to be decreased after exposure (14, 21, and 28 days) to malathion in both treatments (T2 and T3) compared to the control group (T1). Such a decrease may be due to energetic expenditures caused by malathion toxicity. It is possible that fish exposed to chronic stress suffer substrate depletion that leads to a decrease in plasma glucose. The observed decrease in glucose level was to attend energetic expenditures caused by pesticides [41, 42].

A significant decrease in Hb concentration to malathion may be due to less oxygen supply to different tissues, resulting in sluggish metabolic rate and low energy output [25]. The decline in Hb and RBC values is due to the breakdown of iron synthesizing machinery [43]. The damage caused to the intestine by the toxicant may be a reason for impaired iron absorption that led to its deficiency [44].

PCV readings are important in determining the effect of pesticides and in determining the oxygen-carrying capacity of blood [45]. The decreased PCV values are observed when fish stop feeding or become diseased or stressed [46]. In the present study, the distinct decreased level of hemoglobin and PCV after exposure to malathion indicates a hemodilution mechanism due to gill damage or impaired osmoregulation. Hemodilution has been described as a mechanism that reduces the concentration of the stress factor in the circulatory system [47]. Similar findings were found in freshwater fish, *Clarias batrachus* (L.) [27].

During malathion exposure, the decrease in the RBC count may be due to the increase rate of erythrocyte destruction in the hematopoietic organ or due to the inhibition of erythropoiesis, chemosynthesis, or osmoregulatory dysfunction [48]. Due to toxicity, hemopoietic tissue gets affected and becomes unable to release normal RBC in blood circulation [49]. A decrease in the red blood cells could also be the result of internal bleeding caused by the damaged kidney [49]. The significant reduction of RBC might also be due to the hypoxic condition that formed during the treatment, which destroys or decrease the formation of RBC due to the lack of Hb content in the circulatory system [50]. Similar findings were obtained in *Clarias gariepinus* and in *Labeo rohita* by treating them with monocrotophos [51, 52].

Immunological activities and defense mechanisms are usually maintained by WBC [53]. One of the most basic ways to assess the immune system is to explore the changes in WBC [54]. In the present study, WBC significantly increased with increasing the toxicity (concentration or duration-dependent) of malathion as an adaptive measure for the tissue under chemical stress, which also aids in the removal of cellular debris of necrosed tissue at a faster pace. In the presence of foreign substances or under pathological conditions, leukocytosis in fish may be the consequence of direct stimulation of WBC count [55]. The increase in WBC count is also linked to an increase in antibody production, which aids in the recovery of fish that have been exposed to malathion [56]. A significant increase in the WBC was also observed in several studies [57, 58].

In this study, significant (*p* < 0.05) alterations were observed in MCV, MCH, and MCHC with increasing exposure duration of malathion compared to control. This may be due to the fact that these are extremely responsive and can result in flexible changes in the homeostatic system of fish. Fluctuations in these indices are directly proportional to the values of RBC count, hemoglobin concentration, and packed cell volume. The present findings are in line with the earlier reports where the authors also observed alteration in these indices with increasing concentration of toxicant [40, 58]. The increase in MCV is due to the decrease in erythrocyte counts [59]. The observed MCV and MCH level increase in both treatments indicates swelling of RBCs. The release of large red blood cells into the circulation may also lead to an increase in MCV and MCH value [60, 61]. However, the decrease in MCV and MCH value in treatment might be due to a high percentage of immature RBCs in the circulation [58]. Furthermore, in the blood, the large number of undeveloped smaller RBC due to hyperplasia in the erythropoietic sites also results in a higher MCV [62]. The observed rise in MCHC during lethal exposure may be due to genetic spherocytosis [63]. The reduction in MCV, MCH, and MCHC in the blood of *Cyprinus carpio* under Ni stress was reported [64]. Low MCHC indicates a decrease in Hb synthesis. In case of MCHC, a biphasic response was recorded in both the treatments during exposure of malathion and recovery period.

Data on the ability of fish to recover after the removal of pesticides is scanty. During the recovery period, blood glucose, hemoglobin, and hematocrit levels showed partial recovery within 28 days. Similar results were also observed in several studies in which acute exposures of fish organisms to different pesticides were studied [65, 66]. A slight increase in RBC count during recovery compared to a 28-day exposure is probably due to slow recovery from an adverse condition in the fish. The cypermethrin-exposed *Labeo rohita* also showed a similar pattern of recovery following an 80-day cycle of exposure to freshwater [67]. The values of WBC obtained during the recovery period were lower compared to the 28 days of exposure to all concentrations. A significant decreasing trend, observed in the number of WBC, has been reported in *Cyprinus carpi*o exposed to monocrotophos free freshwater [68].

The values of MCV, MCH, and MCHC were affected indirectly because RBC count, Hb level, and PCV values improved and changed in the malathion-free water. Similar findings were observed during the recovery period in several studies [15, 67, 68]. MCV and MCH values were increased after transferring malathion-treated fish into pesticide-free water. Similar observations were recorded in the studies carried out on the toxicity of Furadan (carbofuran 3% g) on hematological, biochemical, and enzymological changes and recovery patterns in *Cyprinus carpio* [53].

## 5. Conclusion

Finally, *B. gonionotus* is somewhat poisonous to malathion. Malathion exposure, even at low concentrations, altered some hematological parameters in *B. gonionotus* and caused stress in the fish. After 28 days of recovery in clean water, fish exposed to malathion had partial recovery. The alterations in hematological parameters may provide early warning signals for the determination of the sublethal toxic level of pesticides. Long-time exposure of fish to pesticides means continuous health hazards for the population. By consuming these fish (toxicated), the human population is now at high risk. So, it is necessary to take precautions in the application of pesticides to protect humans and wildlife.

## Figures and Tables

**Table 1:** Effects of sublethal exposure of malathion on hematology (mean ± SD) at different time intervals in *B. gonionotus*.

Parameter	Treatments	Exposure time (days)				
		1	7	14	21	28
Hemoglobin (g/dL)	T1	13.63 ± 0.52^1a^	12.77 ± 0.77^1a^	13.04 ± 0.66^1a^	12.52 ± 0.46^1a^	12.69 ± 0.49^1a^
	T2	12.97 ± 048^1a^	11.89 ± 0.53^1ab^	10.69 ± 0.4^2bc^	9.97 ± 0.29^2c^	9.71 ± 0.6^2c^
	T3	11.39 ± 0.82^2a^	8.25 ± 0.90^2b^	7.65 ± 0.42^3bc^	6.48 ± 0.54^3cd^	6.03 ± 0.49^3d^

PCV (%)	T1	45.26 ± 3.37^1a^	43.88 ± 4.34^1a^	42.99 ± 4.34^1a^	43.07 ± 4.36^1a^	43.68 ± 4.44^1a^
	T2	43.64 ± 1.67^1a^	39.23 ± 2.20^1ab^	36.04 ± 2.48^1bc^	34.46 ± 1.70^2bc^	31.08 ± 2.11^2c^
	T3	42.18 ± 1.40^1a^	27.38 ± 3.85^2b^	24.06 ± 3.04^2b^	22.38 ± 2.55^3b^	20.3 ± 1.65^3b^

RBCs (×10^6^/mm^3^)	T1	5.72 ± 0.51^1a^	5.79 ± 0.60^1a^	5.87 ± 0.73^1a^	5.75 ± 0.58^1a^	5.69 ± 0.22^1a^
	T2	5.29 ± 0.65^1a^	5.15 ± 0.33^1a^	5.00 ± 0.41^1a^	4.83 ± 0.24^1a^	4.67 ± 0.39^2a^
	T3	4.97 ± 0.34^1a^	4.37 ± 0.55^2a^	4.03 ± 0.11^2ab^	3.18 ± 0.65^2b^	3.17 ± 0.25^3b^

WBCs (×10^4^/mm^3^)	T1	3.19 ± 0.42^3a^	3.02 ± 0.29^2a^	3.10 ± 0.61^2a^	3.12 ± 0.19^3a^	3.03 ± 0.64^2a^
	T2	3.83 ± 0.30^2a^	4.11 ± 0.60^1a^	4.26 ± 0.39^1a^	4.31 ± 0.47^2a^	4.37 ± 0.36^1a^
	T3	4.70 ± 0.56^1a^	5.03 ± 0.15^1a^	5.24 ± 0.07^1a^	5.48 ± 0.24^1a^	5.51 ± 0.30^1a^

MCV (*μ*m^3^)	T1	79.12 ± 6.28^1a^	74.49 ± 4.72^1a^	73.55 ± 5.55^1a^	74.90 ± 12.80^1a^	76.76 ± 5.20^1a^
	T2	82.49 ± 6.99^1a^	76.17 ± 1.79^1ab^	72.07 ± 1.58^1ab^	71.31 ± 2.30^1b^	66.55 ± 5.06^2b^
	T3	84.86 ± 3.44^1a^	62.65 ± 4.15^2a^	59.90 ± 9.11^2a^	70.37 ± 17.93^1a^	64.02 ± 0.68^3a^

MCH (pg)	T1	24.03 ± 2.78^1a^	22.15 ± 1.03^1a^	22.44 ± 2.62^1a^	22.01 ± 2.79^1a^	22.31 ± 1.00^1a^
	T2	24.72 ± 2.17^1a^	23.14 ± 0.90^1ab^	21.43 ± 1.02^1ab^	20.69 ± 1.40^1b^	20.83 ± 0.69^1b^
	T3	22.93 ± 1.07^1a^	18.97 ± 1.30^2a^	18.98 ± 0.60^2a^	21.02 ± 4.08^1a^	19.10 ± 1.96^2a^

MCHC (%)	T1	30.22 ± 2.15^1a^	29.28 ± 2.42^1a^	30.47 ± 1.75^1a^	29.31 ± 3.04^1a^	29.30 ± 3.13^1a^
	T2	29.72 ± 0.16^1a^	30.33 ± 0.65^1a^	29.72 ± 1.12^1a^	29.01 ± 1.67^1a^	31.29 ± 1.46^1a^
	T3	27.01 ± 1.78^1a^	30.25 ± 1.03^1a^	32.42 ± 5.90^1a^	29.49 ± 5.66^1a^	29.86 ± 3.35^1a^

Values with different numeric superscripts differ significantly (*p* < 0.05) between concentrations within the duration. Values with different letter superscripts differ significantly (*p* < 0.05) between duration within the concentration. All values are expressed as mean ± SD.

**Table 2:** Ability of fish to recover at pesticide-free water for a period of 28 days (T2: 25% of LC_50_; T3: 50% of LC_50_).

Parameter	Control	Treatments	After 28 days of exposure	Exposure time to pesticide-free water in days (recovery rates)				
				1	7	14	21	28
Hb (g/dL)	12.93 ± 0.58^1a^	T2	9.71 ± 0.60^2c^	9.86 ± 0.89^2a^ (1.54%)	10.07 ± 0.18^2a^ (3.71%)	10.55 ± 0.76^2a^ (8.65%)	10.94 ± 0.99^2a^ (12.66%)	11.36 ± 0.63^2a^ (16.99%)
		T3	6.03 ± 0.49^3d^	6.09 ± 0.91^3a^ (0.99%)	6.17 ± 0.98^3a^ (2.32%)	6.42 ± 0.72^3a^ (6.47%)	6.65 ± 0.49^3a^ (10.28%)	6.85 ± 0.34^3a^ (13.59%)

PCV (%)	47.95 ± 0.49^1a^	T2	31.08 ± 2.11^2c^	31.63 ± 1.13^2c^ (1.76%)	32.58 ± 1.50^2bc^ (4.83%)	33.60 ± 0.86^2abc^ (8.11%)	34.71 ± 1.11^2ab^ (11.68%)	35.95 ± 0.41^2a^ (15.67%)
		T3	20.3 ± 1.65^3b^	20.53 ± 0.51^3b^ (1.13%)	21.08 ± 0.95^3ab^ (3.84%)	21.67 ± 0.65^3ab^ (6.75%)	22.04 ± 1.12^3ab^ (8.57%)	22.88 ± 0.51^3a^ (12.71%)

RBCs (×10^6^/mm^3^)	5.76 ± 0.58^1a^	T2	4.67 ± 0.39^2a^	4.72 ± 0.37^1a^ (1.07%)	4.79 ± 0.33^1a^ (2.56%)	4.87 ± 0.51^1a^ (4.28%)	4.96 ± 0.35^1a^ (6.20%)	5.07 ± 0.45^1a^ (8.56%)
		T3	3.17 ± 0.25^3b^	3.20 ± 0.62^2a^ (0.95%)	3.23 ± 0.39^2a^ (1.89%)	3.26 ± 0.38^2a^ (2.83%)	3.34 ± 0.71^2a^ (5.36%)	3.39 ± 0.33^2a^ (6.94%)

WBCs (×10^4^/mm^3^)	3.09 ± 0.43^2a^	T2	4.37 ± 0.36^1a^	4.29 ± 0.57^12a^ (−1.83%)	4.18 ± 0.35^2a^ (−4.34%)	4.06 ± 0.45^12a^ (−7.09%)	3.99 ± 0.14^2a^ (−8.69%)	3.71 ± 0.43^2a^ (−15.10%)
		T3	5.51 ± 0.30^1a^	5.46 ± 0.47^1a^ (−0.91%)	5.39 ± 0.61^1a^ (−2.17%)	5.21 ± 0.54^1a^ (−5.44%)	5.14 ± 0.46^1a^ (−6.71%)	5.05 ± 0.48^1a^ (−8.34%)

MCV (*μ*m^3^)	82.67 ± 3.40^1a^	T2	66.77 ± 5.06^2b^	67.27 ± 5.02^1a^ (0.75%)	68.09 ± 2.83^2a^ (1.97%)	69.56 ± 5.87^12a^ (4.17%)	70.34 ± 7.11^1a^ (5.34%)	71.36 ± 6.10^12a^ (6.87%)
		T3	64.02 ± 0.68^3a^	66.76 ± 15.76^1a^ (4.27%)	65.85 ± 7.08^2a^ (2.85%)	67.15 ± 6.56^2a^ (4.88%)	68.50 ± 15.01^1a^ (6.99%)	67.98 ± 5.33^2a^ (6.18%)

MCH (pg)	24.63 ± 1.32^1a^	T2	20.83 ± 0.69^1b^	21.10 ± 3.49^1a^ (1.29%)	21.09 ± 1.56^12a^ (1.24%)	21.78 ± 1.60^12a^ (4.56%)	22.22 ± 3.37^1a^ (6.67%)	22.48 ± 1.28^12a^ (7.92%)
		T3	19.10 ± 1.96^2a^	19.60 ± 4.41^1a^ (2.62%)	19.18 ± 2.78^2a^ (0.41%)	19.72 ± 0.59^2a^ (3.25%)	20.64 ± 4.55^1a^ (8.06%)	20.30 ± 1.22^2a^ (6.28%)

MCHC (%)	32.21 ± 0.27^1a^	T2	31.29 ± 1.46^1a^	31.25 ± 3.61^1a^ (−0.13%)	30.95 ± 1.29^1a^ (−1.08%)	31.39 ± 2.03^1a^ (0.32%)	31.48 ± 1.87^1a^ (0.61%)	31.59 ± 1.80^1a^ (0.96%)
		T3	29.86 ± 3.35^1a^	29.71 ± 4.70^1a^ (−0.50%)	29.16 ± 3.35^1a^ (−2.34%)	29.56 ± 2.48^1a^ (−1.00%)	30.13 ± 0.73^1a^ (0.90%)	29.91 ± 0.86^1a^ (0.17%)

Values with different numeric superscripts differ significantly (*p* < 0.05) between concentrations within the duration. Values with different letter superscripts differ significantly (*p* < 0.05) between duration within the concentration. All values are expressed as mean ± SD. Values within parenthesis indicate recovery rates.

## Data Availability

The data used to support the findings of this study are included within the article.
